# Development of a Gold Nanoparticle Vaccine against Enterohemorrhagic Escherichia coli O157:H7

**DOI:** 10.1128/mBio.01869-19

**Published:** 2019-08-13

**Authors:** Javier I. Sanchez-Villamil, Daniel Tapia, Alfredo G. Torres

**Affiliations:** a Department of Microbiology and Immunology, University of Texas Medical Branch, Galveston, Texas, USA; b Department of Pathology, University of Texas Medical Branch, Galveston, Texas, USA; UT Southwestern Med Center Dallas; University of Michigan; University of Virginia

**Keywords:** *Escherichia coli*, O157:H7, diarrhea, nanovaccines, pathogenic *Escherichia*, vaccines

## Abstract

Here we exploit the natural properties of a synthetic nanoparticle (NP) scaffold as a subunit vaccine against enterohemorrhagic Escherichia coli (EHEC). Two EHEC-specific immunogenic antigens, namely, LomW and EscC, either alone or in combination, were covalently linked on the surface of gold nanoparticles (AuNPs) and used to immunize mice prior to challenge with EHEC O157:H7 strain 86-24. LomW is a putative outer membrane protein encoded in bacteriophage BP-933W, while EscC is a structural type III secretion system protein which forms a ring in the outer membrane. The resulting AuNP preparations, AuNP-LomW and AuNP-EscC, showed that the nanoparticles were able to incorporate the antigens, forming stable formulations that retained robust immunogenicity *in vivo* after subcutaneous immunization. When administered subcutaneously, AuNP-LomW or AuNP-EscC or a combination containing equivalent amounts of both candidates resulted in higher IgG titers in serum and secretory IgA titers in feces. The serum IgG titers correlated with a significant reduction in EHEC intestinal colonization after 3 days postinoculation. In addition, we showed that serum from antigen-coated AuNP-immunized mice resulted in a reduction of adherence to human intestinal epithelial cells for EHEC, as well as for two other E. coli pathotypes (enteropathogenic E. coli [EPEC], encoding EscC, and enteroaggregative E. coli [EAEC], encoding LomW). Further, the serum had antigen-specific bactericidal properties, engaging the classical complement pathway. Overall, our results demonstrate the immunogenicity and stability of a novel nanovaccine against EHEC. These results also strengthen the prospect of development of a synthetic nanoparticle vaccine conjugated to E. coli antigens as a promising platform against other enteric pathogens.

## Abstract (precis)

Enterohemorrhagic E. coli O157:H7 is a human pathogen and the causative agent of diarrhea and hemorrhagic colitis, which can progress to hemolytic uremic syndrome. These complications represent a serious global public health problem that requires laborious public health interventions and safety control measures to combat recurrent outbreaks worldwide. Today, there are no effective interventions for the control of EHEC infections, and, in fact, the use of antibiotics is counterindicated for EHEC disease. Therefore, a viable alternative for the prevention of human infections is the development of vaccines; however, no such vaccines are approved for human use. In this study, we developed a novel gold nanoparticle platform which acts as a scaffold for the delivery of various antigens, representing a nanovaccine technology which can be applied to several disease models.

## INTRODUCTION

Diarrheal diseases are among the leading causes of morbidity and mortality, especially among children under the age of 5 years. A significant proportion of diarrheal disease is caused by diarrheagenic Escherichia coli (1). Due in part to their pathogenic mechanisms, clinical symptomologies, and virulence factors, pathogenic E. coli strains are classified into different pathotypes (2, 3). Enterohemorrhagic E. coli (EHEC), in particular, serotype O157:H7, is a unique human pathogen which is associated with severe complications and high mortality compared to other pathotypes. In addition, the primary reservoir for EHEC includes ruminants and the main route of infection involves ingestion of contaminated food products which often result in outbreaks. Infections caused by EHEC can range from acute self-resolving diarrheal episodes to hemorrhagic colitis, which can progress to hemolytic-uremic syndrome (HUS) (2, 3). The production of Shiga toxins (Stx) is one of the defining characteristics of EHEC O157:H7 pathogenesis and the key virulence factor associated with HUS. However, the first step in the pathogenic process of EHEC is adherence to intestinal epithelial cells (IECs) (1, 4). To colonize the intestinal mucosa, EHEC O157:H7 is known to utilize a myriad of pili and/or fimbriae to attach to IECs (4). Subsequently, EHEC binding results in an intimate attachment to the surface of IECs and injection of virulence factors by the use of its type III secretion system (TTSS) apparatus. The translocated virulence factors are responsible for the formation of histological changes known as attaching and effacing (A/E) lesions on epithelial cells. These lesions are characterized by the disruption of the microvilli and the accumulation of actin below the site of bacterial adherence to create a “cup-like structure” (2). The genetic factors necessary for the biogenesis of a functional T3SS and of many of its effectors are harbored by a pathogenicity island called the locus of enterocyte effacement (LEE) (5).

Despite the pathogenic processes and the likelihood of causing severe disease and sequelae in humans, there are no specific treatment options available to combat EHEC infections. In fact, the conventional use of antibiotics only aggravates the disease because it promotes and exacerbates the expression of gene products encoding the Stx toxins (Stx1 and Stx2) which are located within a lambdoid phage. This process induces bacterial lysis, allowing the release and dissemination of Stx into the intestine and other organs (6). Therefore, the development of new strategies to control infections caused by EHEC is necessary. A viable alternative to prevent infection is the development of vaccines; however, there are no approved human vaccines against this pathogen. The advances in vaccine development have included different platforms and approaches such as live attenuated, whole-cell killed, subunit, toxoid, polysaccharide, and polysaccharide-protein conjugates (7, 8). Previous studies have focused on the development of vaccines based on proteins encoded in the LEE pathogenicity island, such as the intimin adhesin and the T3SS component EspA (9), as well as other peptides (10), different fusion constructs containing subunits A and B of Stx1 and Stx2 (11), or avirulent bacterial ghost cells derived from EHEC O157:H7 (12). These different vaccine candidates have shown variable success in various *in vivo* murine models of infection.

Recently, gold nanoparticles (AuNPs) have received attention due to their potential use over traditional vaccine platforms (13). The location of AuNPs in lymphoid tissues and cells (14, 15), their capacity of coupling to a variety of biomolecules (16–18), their stability (19, 20), and their safety (13, 16) (essential for the development and synthesis of vaccines) provide a multifaceted focus for the design of this new vaccine platform. Previously, we identified two immunogenic protein candidates specific for EHEC using a reverse vaccinology approach. These candidates were tested using a DNA vaccine vector and demonstrated differing degrees of protection (21–23). Therefore, the objective of this study was to conjugate these two EHEC O157:H7 antigen candidates to the AuNP platform and assess whether the nanovaccines can induce protective responses *in vitro* and *in vivo*. Our work proposes to bridge the properties of subunit vaccination with those of synthetic nanomaterials to enhance immune responses to vaccines against pathogenic E. coli. We have developed a multidisciplinary pipeline for the identification of antigens and for efficient delivery of nanovaccines that can be used against other pathogenic bacterial organisms.

## RESULTS

### Purification of immunogenic proteins and coupling of AuNP vaccines.

**FIG 1:**
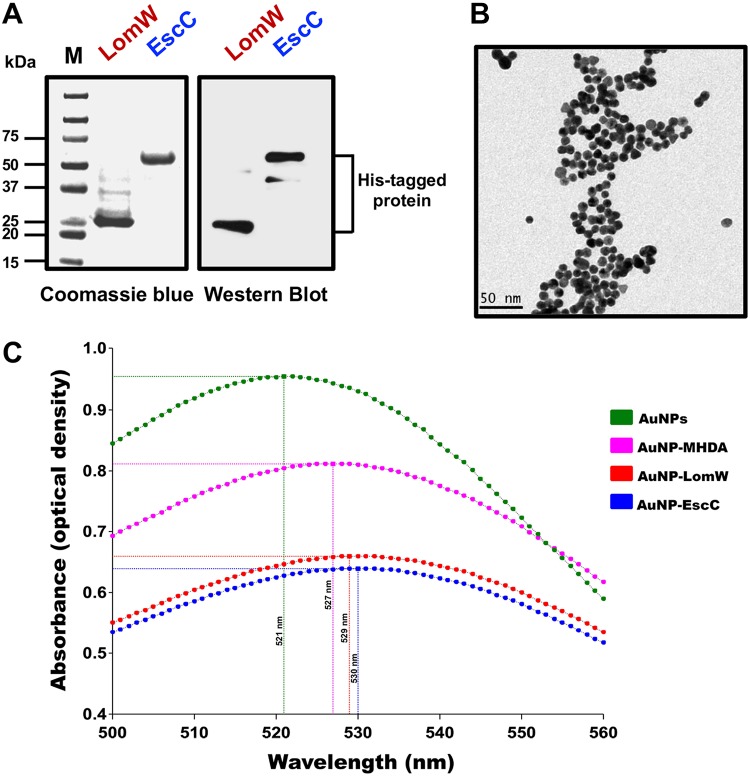
Purification and characterization of AuNP platform after conjugation to LomW and EscC. (A) Coomassie-stained gel and Western blot with anti-His showing the purity of LomW (28 kDa) and EscC (56 kDa). Purification of His-tagged recombinant protein from E. coli BL21 was performed using Co^2+^ affinity chromatography. (B) Transmission electron micrograph of bare AuNPs showing consistent sizes and shapes. Bar, 50 nm. (C) Absorption spectra of nonconjugated AuNPs (green), AuNPs with MHDA linker (pink), and AuNPs after covalent conjugation with LomW (red) and EscC (blue). The displacement shows a red shift after successful conjugation of protein.

Using information from a comparative bioimmunoinformatic analysis performed in our laboratory, two O157:H7-specific candidates were selected based on their predicted immunogenicity, bacterial surface location, and human major histocompatibility complex (MHC) allele coverage and because they were previously demonstrated to be protective when used as DNA vaccines (21, 23). To use these proteins as subunit vaccine candidates, we selected a gold nanoparticle (AuNP) platform to display the antigens and to immunize mice subcutaneously (s.c.). AuNPs have gained interest as vaccine scaffolds because they have been used to incorporate, stabilize, and increase the immunogenicity of subunit vaccine candidates (15). We purified two proteins, LomW (933W phage-encoded outer membrane protein) and EscC (type III secretion system structural protein) under native conditions using affinity chromatography to allow efficient conjugation onto the AuNP surface. The proteins were tested for endotoxin presence, and no levels of endotoxin were found with a limit of detection of 0.1 endotoxin units (EU)/ml. The histidine (His)-tagged proteins were visualized by Coomassie staining and Western blotting (Fig. 1A). To consistently synthesize spherical 15-nm-diameter AuNPs, the Turkevich method was used (24), and the resulting particles were visualized using transmission electron microscopy (TEM) (Fig. 1B). The protein candidates were immobilized on the AuNP surface by adding 16-mercaptohexaundecanoic acid (MHDA), which is a small linker with a thiol group that readily binds AuNP and possesses a carboxylic acid on the distal end used to covalently attach biomolecules (25). We confirmed the conjugation of these proteins to MHDA-coupled AuNPs by UV-visible light (UV-Vis) spectroscopy as seen by the wavelength displacement to 529 nm and 530 nm upon addition of LomW and EscC, respectively (Fig. 1C). This red shift (2 nm and 3 nm for LomW and EscC, respectively) after the addition of proteins onto the AuNPs allowed us to characterize the stable coupling of AuNP-protein platform for subsequent *in vivo* studies.

### Reduced gastrointestinal colonization in animals immunized with AuNP-EscC and AuNP-LomW.

**FIG 2:**
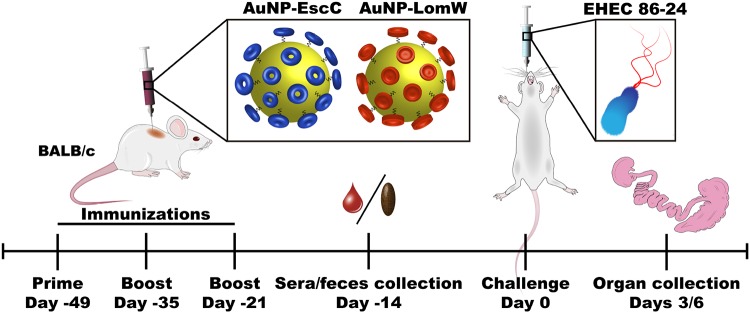
Timeline of vaccination schedule. The figure shows a schematic of vaccination of BALB/c mice and challenge with EHEC O157:H7 strain 86-24, followed by blood, feces, and organ collection.

**FIG 3:**
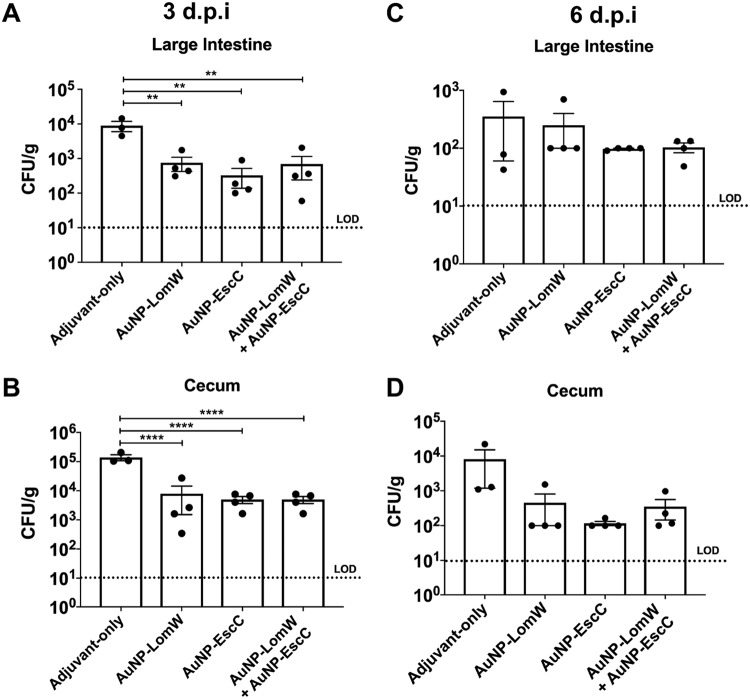
Reduced bacterial colonization from AuNP-protein-vaccinated mice. After challenge via gavage with 3 × 10^9^ CFU of EHEC 86-24, large intestines (top panels) and ceca (bottom panels) from BALB/c mice (*n* = 8) were collected at 3 (A and B) and 6 (C and D) days postinfection. Organs were processed, diluted, and plated on MacConkey agar plates for bacterial enumeration. Bacterial load was determined per gram of tissue, and representative panels for colonization are shown in log scale. The bacterial limit of detection (LOD) was 10 CFU/organ. All colonization data are shown as means ± standard errors of the means (SEM) of results determined per group. Significant differences in organ colonization were determined via one-way ANOVA followed by Tukey’s *post hoc* test (*, *P* < 0.05; **, *P* < 0.001; ***, *P* < 0.0001).

After confirming the proper coupling of stable AuNP-protein conjugates, we assessed the protective efficacy and multivalency of AuNP vaccines (nanovaccines) in an *in vivo* murine model of E. coli infection. Mice were immunized subcutaneously (s.c.) using the scheme displayed in Fig. 2. BALB/c mice received three immunizations in 2-week intervals that contained a total of 10 μg of protein conjugated onto AuNPs for each vaccination and that were further combined with adjuvants. A group of mice received a combination formulation containing approximately 5 μg each of the two AuNP-conjugated candidates. Serum and fecal samples were collected 2 weeks after the last immunization for evaluation of humoral responses. Mice were then challenged 3 weeks after the last immunization with 3 × 10^9^ CFU of EHEC O157:H7 (strain 86-24). Stomach acidity was reduced 2 h prior to infection by administering cimetidine (50 mg/kg of body weight). At 3 and 6 days postchallenge, we collected samples from the large intestine and cecum and evaluated bacterial colonization. Immunization with AuNP-LomW or with AuNP-EscC or with AuNP-combination (AuNP-LomW plus AuNP-EscC) resulted in a significant reduction in colonization in both the cecum and large intestine at 3 days postimmunization (Fig. 3). In all three immunization groups, there was a remarkable reduction in colonization (limit of detection [LOD] of 10 CFU/organ) in both the large intestine (Fig. 3A) and the cecum (Fig. 3B) at 3 days postinfection (dpi). We saw a similar pattern of reduced colonization at 6 dpi (see Fig. 3C and D), but the differences were smaller because the animals had begun to clear the wild-type bacteria from the gastrointestinal (GI) tract (see Fig. S1 in the supplemental material). These results confirmed the vaccine properties of our AuNP-protein immunization in the reduction of EHEC colonization *in vivo*.

10.1128/mBio.01869-19.1 FIG S1 EHEC O157:H7 bacterial counts in feces after infection. Levels o fexcretion of EHEC O157:H7 were determined as CFU counts per gram of feces for the first 6 days postchallenge. The results are expressed as means ± standard errors of the means (SEM) of results from eight mice in each group. Fecal pellets were collected in preweighed 1-ml PBS tubes and weighted after sample collection. After processing and dilution in PBS, fecal samples were plated on MacConkey agar plates and incubated at 37°C prior to counting. Download FIG S1, TIF file, 2.4 MB. Copyright © 2019 Sanchez-Villamil et al. 2019 Sanchez-Villamil et al. This content is distributed under the terms of the Creative Commons Attribution 4.0 International license.

### AuNP-protein immunization resulted in robust humoral mediated immunity.

**FIG 4:**
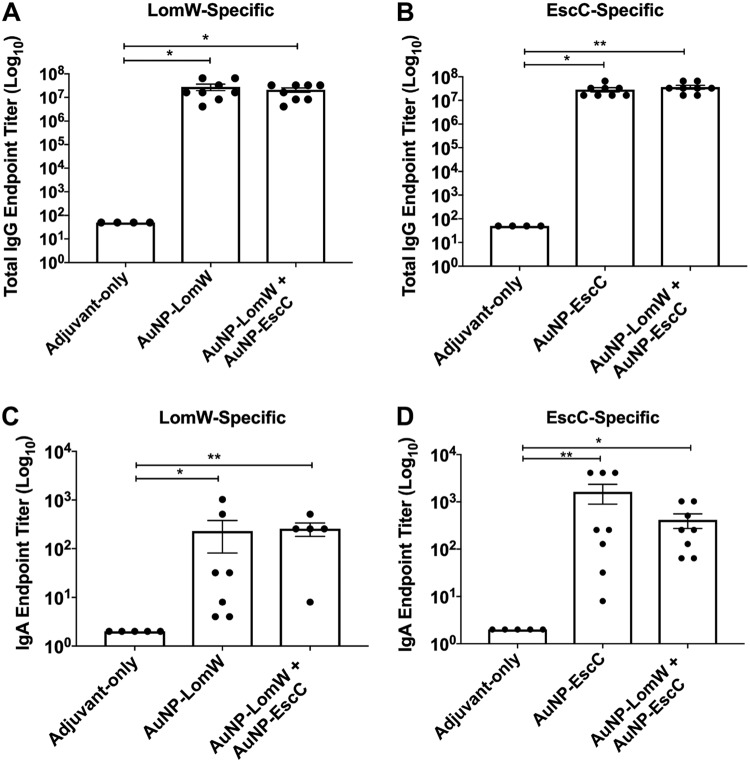
Antibody responses after AuNP-protein immunization. (A) LomW-specific total IgG titers and (C) secretory IgA titers, as well as (B) EscC-specific total IgG antibody responses and (D) secretory IgA titers, were assessed via ELISA, with endpoint titers defined as twice the standard deviation (SD) of the levels measured for naive sera. Sera and fecal samples were collected 2 weeks after the last immunization. Samples taken from the mice immunized with the combination formulation were used to assay anti-LomW and anti-EscC antibody titers. All antibody data are expressed as mean ± SEM of results from *n* = 8 mice per group. Significant differences in IgG and IgA titers were determined via one-way ANOVA followed by Tukey’s *post hoc* test (*, *P* < 0.05; **, *P* < 0.001; ***, *P* < 0.0001).

To evaluate the immunogenicity of the AuNP-LomW and AuNP-EscC nanovaccines, we evaluated the production of protein-specific total IgG and secretory IgA (sIgA) responses in immunized mice. Using the serum and fecal samples collected after the last immunization, we compared the total IgG endpoint titers from the AuNP-LomW, AuNP-EscC, and combination formulations against each antigen. The IgG endpoint titers against LomW and EscC were significantly higher for all three vaccine formulations than were seen with the group of mice immunized with adjuvant only (Fig. 4A and B). The levels of IgG demonstrate the ability of AuNP-protein vaccination to elicit strong systemic antigen-specific IgG responses. Likewise, the total sIgA endpoint titers were significantly higher for LomW and EscC in both vaccine groups (Fig. 4C and D). The levels of sIgA confirm that AuNP-protein immunization elicited strong mucosal IgA responses irrespective of the vaccination route. These results demonstrate that AuNP-LomW, AuNP-EscC, and AuNP-combination can elicit strong IgG and sIgA responses. Next, we evaluated the functionality of these antibody responses during *in vitro* experiments by determining whether the serum antibodies were able to mediate inhibition of the adherence and establishment of infection.

### Antibodies from AuNP immunization decreased bacterial adherence to intestinal epithelial cells.

**FIG 5:**
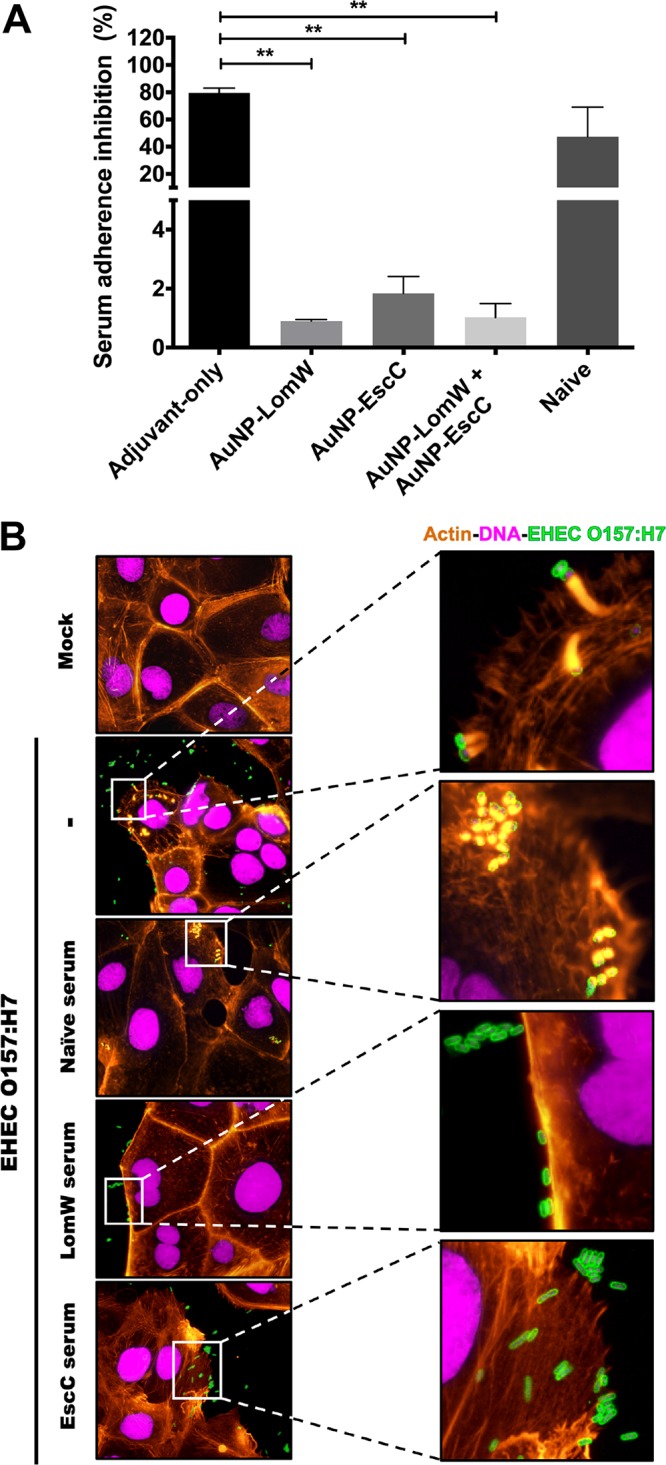
Serum from AuNP-protein-immunized mice reduces EHEC 86-24 adherence and pedestal formation on Caco-2 intestinal epithelial cells. EHEC 86-24 bacterial cells (1 × 10^7^ CFU) were incubated in the presence of immunized serum (10% final volume) from AuNP-LomW, AuNP-EscC, and AuNP-LomW plus AuNP-EscC for 1 h at 37°C. Serum from naive mice or mice subjected to adjuvant-only immunization served as controls. After incubation, bacteria were used to infect Caco-2 cells for 2 h. (A) After infection, cell monolayers were washed, detached, and diluted to enable enumeration of adhered bacteria. All adherence data are expressed as mean ± SEM of results from two independent experiments using sera from *n* = 8 mice per group. Significant differences were determined via one-way ANOVA followed by Tukey’s *post hoc* test (*, *P* < 0.05; **, *P* < 0.001; ***, *P* < 0.0001). (B) Fluorescence microscopy analysis of Caco-2 cells after EHEC infection in the presence of immune serum (from vaccinated groups). After infection, cells were fixed and stained with phalloidin-rhodamine (actin) or DAPI (bacteria and nuclei) and examined for EHEC by immunofluorescence (anti-EHEC primary antibody conjugated to FITC). Panels on the right present magnifications of the images on the left. The magnified views show the formation of actin pedestals and the bacterial adherence pattern.

The capacity of antibodies to neutralize pathogens in mucosal surfaces may be important for host protection, especially against enteric bacteria. To assess the functionality of these antibodies, we tested whether serum was able to inhibit bacterial adherence to human intestinal epithelial cells (IECs; Caco-2). Using 10% serum from AuNP-vaccinated mice, we quantified adherence of EHEC to IECs and detected a reduction of approximately 98% when EHEC (multiplicity of infection [MOI] = 10) was incubated prior to infection with AuNP-LomW or with AuNP-EscC or with the combination sera (Fig. 5A). In contrast, serum from animals that were vaccinated with the adjuvant-only control or with serum from naive animals had about a 20% reduction in bacterial adherence (Fig. 5A). To calculate levels of adhered bacteria, the different samples were all compared to the input inoculum.

To further analyze the neutralizing capacity of the antibodies found in the immune serum, an *in vitro* assay was performed to evaluate adherence of EHEC to Caco-2 cells. One of the hallmark pathogenic mechanisms operating during EHEC infection is that of the intimate adherence to the epithelial surface and the formation of polymerized actin pedestals (2, 4). To visualize bacterial adherence and the rearrangement of actin cytoskeleton, Caco-2 cells were labeled with fluorescent actin stain (FAS), nuclei were labeled with DAPI (4′,6-diamidino-2-phenylindole), and a commercial antibody was used to detect EHEC. In the case of EHEC-infected cells, it was possible to observe, using fluorescence microscopy, a diffuse adherence pattern and the accumulation of polymerized actin beneath the site of bacterial attachment (Fig. 5B). In contrast, immune serum from vaccinated mice inhibited the formation of actin pedestal structures and reduced the number of bacteria attached to host cells (Fig. 5B). These results corroborate our quantitative analysis and further demonstrate a reduction in bacterial adherence in the presence of immune serum (Fig. 5A). Overall, we found that serum from AuNP-vaccinated mice significantly reduced the attachment of EHEC to IECs, thereby preventing the formation of actin pedestals.

**FIG 6:**
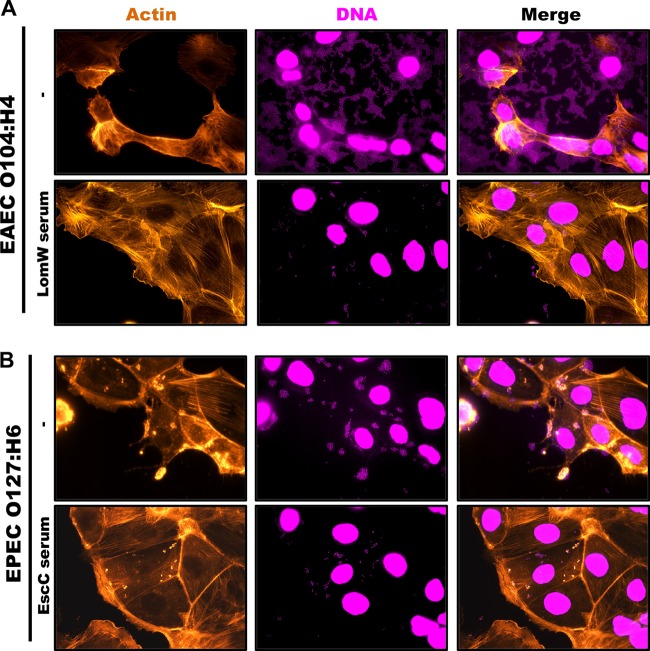
A cross-protective effect of immune serum from AuNP-protein-vaccinated mice reduces adherence and cytotoxic effect induced by EAEC or EPEC on Caco-2 intestinal epithelial cells. Infection of Caco-2 cells was carried out as described for Fig. 5. (A) EAEC O104:H4 was incubated in the presence or absence of AuNP-LomW immune serum for 1 h. After incubation, bacteria were used to infect Caco-2 cells for 2 h, and the cells were stained for fluorescence microscopy. (B) EPEC O127:H6 was incubated in the presence or absence of AuNP-EscC immune serum prior to infection in Caco-2 cells. After infection, cells were fixed and stained as described in the legend to Fig. 5.

We then tested whether the serum from vaccinated mice was able to block the adherence of other pathotypes which share some of the antigens coupled to the nanovaccines. Enteroaggregative E. coli (EAEC) O104:H4 is a hybrid strain that was associated with numerous HUS cases during the German outbreak in 2011 and which possesses EAEC virulence factors but also encodes Shiga toxin (26). On the other hand, enteropathogenic E. coli (EPEC), like EHEC, carries the LEE pathogenicity island which encodes the T3SS components required for A/E lesion formation (2, 3). A bioinformatic comparison confirmed that EscC and LomW of EHEC are highly conserved between EPEC (EscC) and EAEC (LomW) (Table 1). Therefore, we tested whether antibodies against these proteins conferred cross-protective antiadhesive properties *in vitro* against other pathotypes. A characteristic aggregative adherence pattern in a stack-brick conformation and a cytotoxic effect(s) represented by contraction of the actin cytoskeleton followed by detachment from the substratum are associated with EAEC pathogenesis (2, 3). We observed that these adhesive properties were affected during EAEC infection in the presence of LomW-specific serum (Fig. 6A). In the case of EPEC, infection is characterized by a localized adherence pattern leading to the establishment of microcolonies and subsequent formation of actin pedestals. Unlike EHEC, where complete abolishment of the formation of actin pedestals was observed, EscC-specific serum partially reduced bacterial adherence and the formation of pedestals (Fig. 6B). These results indicate that (i) a reduction in the adherence for both pathotypes was achieved and (ii) the presence of LomW–specific antibodies can prevent aggregative adherence and cytotoxicity during EAEC infection, while EscC-specific antibodies reduced adherence and to a lesser extent the formation of pedestals during EPEC infection.

**TABLE 1:** Homology of EHEC^*a*^ EscC and LomW among EPEC and EAEC/EHEC pathotypes

Pathotype	EscC	LomW	% homology/identity to EHEC O157:H7 strain 86-24
EPEC O127:H6	✓		100/99.8
EAEC/EHEC O104:H4		✓	100/100

a O157:H7 strain 86-24.

### Bactericidal activity of AuNP-immunized serum is antigen specific and involves the classical complement pathway.

**FIG 7:**
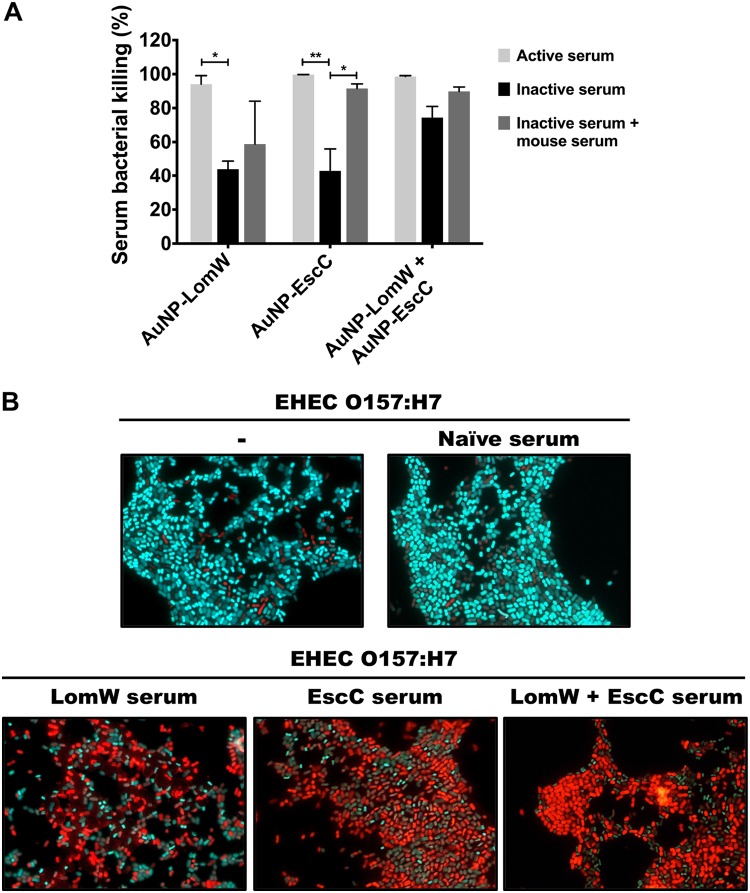
The serum bactericidal activity of immune serum from AuNP-protein-vaccinated mice is antigen dependent. EHEC strain 86-24 (1 × 10^7^ CFU) was incubated with 10% active, inactive, and inactive plus exogenous complement source. Bacteria were incubated in the presence of serum using 10% serum for 1 h at 37°C. (A) Viable CFU counts were determined by plating on LB agar plates. Bacterial killing was normalized using bacteria surviving after exposure to adjuvant-only serum. Data are expressed as means ± SEM of results from two independent experiments using sera from *n* = 8 mice per group. Significant differences were determined via one-way ANOVA followed by Tukey’s *post hoc* test (*, *P* < 0.05; **, *P* < 0.001; ***, *P* < 0.0001). (B) LIVE/DEAD *Bac*Light assay using SYTO9 to stain live bacteria (cyan) and propidium iodide to stain dead bacteria (red). EHEC strain 86-24 (1 × 10^7^ CFU) was incubated in the presence of AuNP-immune and naive serum for 1 h at 37°C prior to staining.

We then evaluated whether the serum from vaccinated mice had bactericidal activity. To test the activity by complement-mediated killing, we quantified the percentage of EHEC killing in the presence of active or inactive serum and of inactive serum supplemented with a source of complement. In the presence of active serum from the three AuNP-immunized mouse groups, we observed almost complete killing but did not see a significant difference in the percentages of bacterial killing between the 3 groups. In contrast, we noticed a significant reduction in the level of bacterial killing with the inactivated AuNP-LomW and AuNP-EscC immunized serum (Fig. 7A). More interestingly, the bacterial killing effect was restored for the AuNP-EscC inactivated serum when an exogenous complement source was added, and to some extent, for the serum of AuNP-LomW and of the AuNP combination (Fig. 7A). To corroborate that complement-mediated killing was the mechanism used to damage the integrity of the bacterial membrane and achieve bacterial clearance, we used a LIVE/DEAD *Bac*Light assay to monitor bacterial viability as a function of the membrane integrity. SYTO9 is a green fluorescent dye that can enter all bacterial cells, whereas propidium iodide (PI) is a red dye that enters bacteria only when the membrane is damaged. We observed an increase in the number of red-stained bacterial cells in the presence of sera from the experiments using AuNP-LomW, AuNP-EscC, and AuNP combination, indicating a loss of membrane integrity (Fig. 7B). In contrast, the majority of untreated EHEC, or of those bacteria treated with naive serum, showed green fluorescent signal (Fig. 7B). This suggests that the bactericidal activity seen with the serum of AuNP-proteins was antigen specific and supports the idea of the engagement of the classical complement pathway as a mechanism of bacterial killing *in vitro*.

## DISCUSSION

The current interest in AuNPs for biomedical applications is a result of their natural properties, including stabilization of antigens and enhancement of uptake by antigen-presenting cells (25, 27). The use of AuNPs has been exploited for the delivery of antigens in several models, including in viral diseases and cancer (19, 25, 27–29). However, there are few reports of AuNP functionalization with bacterial antigens (28, 30). We previously used AuNPs for the construction of nanovaccines against other bacterial pathogens (31–33). Therefore, we sought to exploit the natural properties of AuNPs by coupling two EHEC O157:H7-specific antigens and assessing their protective properties both *in vivo* and *in vitro*. Both LomW and EscC were identified by an extensive bioimmunoinformatic analysis and were predicted to contain several immunogenic epitopes, and our laboratory has previously shown that they can provide some level of protection against EHEC colonization when delivered as a DNA vaccine (21–23). Therefore, we decided to investigate whether we could use the AuNP platform to potentiate the delivery of these antigenic protein candidates and to increase their protective effect. On the basis of our prior experience, we were able to demonstrate that AuNPs form stable scaffolds capable of conjugating different proteins (LomW and EscC) on their surface, allowing stabilization and efficient antigen delivery.

As extensively demonstrated, EHEC O157:H7 colonization onto intestinal epithelial cells is characterized by the formation of tight interactions with epithelial cells, also known as attaching and effacing (A/E) lesions (3). In recent years, EHEC had not received much attention for the development of vaccine candidates because attention had refocused to other pathogenic E. coli strains (7, 8). Nonetheless, studies examining vaccine candidates against EHEC in cattle have focused on various virulence factors, including colonization factors, T3SS structural proteins, or Shiga toxin B (StxB) (34, 35). Although promising, these attempts have shown differing degrees of success. In the present study, we observed a significant reduction in EHEC colonization to the large intestine and cecum of mice that were immunized with AuNP-LomW, AuNP-EscC, and the combination nanovaccine. These results indicate that AuNP-protein immunization elicits a protective immune response capable of neutralizing EHEC, thus preventing the colonization of the mouse GI tract. A previous study highlighted the importance of outer membrane proteins as immunogenic antigens recognized by HUS patients and with antibodies present in their sera (36). Among the proteins that were identified to be reactive in HUS patient sera are the flagella protein (FliC) and outer membrane structural components (OmpF, OmpC), all of them displaying both IgG reactivity and IgA reactivity (36). It is reasonable to propose that, like LomW and EscC, other surface-exposed proteins might be reactive during disease progression; thus, a nanovaccine intervention that elicits both systemic and mucosal antibodies might prevent the development of HUS.

The function of the LomW protein in EHEC pathogenesis has not been characterized, but under conditions of expression, the protein is predicted to be translocated to the bacterial outer membrane as a component of the 933W phage machinery, a genetic region that also encodes the Shiga toxin genes (37). The presence of LomW on the bacterial outer membrane needs further investigation, but if this occurs during infection, the antigen becomes an ideal target for vaccination because it can be readily recognized by antibodies as demonstrated in our experiments. On the other hand, EscC is a structural protein component of the type III secretion system (T3SS), known to participate in the formation of a ring on the bacterial outer membrane (38). The reduction of EHEC adherence to IECs in the presence of EscC-specific antibodies strongly suggests that such inhibition interferes with early events prior to intimate attachment. Previously, it was demonstrated that vaccination with structural T3SS components reduced bacterial fecal shedding (39). Our data confirm those results, but, in addition, we now provide evidence demonstrating that serum can block the formation of actin pedestals on the surface of intestinal cells, which represents a direct effect on the pathogenic mechanism of EHEC and, potentially, other A/E lesion-forming pathogens. In addition, it has been demonstrated that antibodies that inactivate the T3SS inhibit the injection of effector molecules that mediate other bacterial pathogenic mechanisms (39). The absence of actin pedestals in the presence of antigen-specific serum confirms the inhibition of an important pathogenic EHEC mechanism. Although we do not know the mechanism mediating the anti-LomW effect, our results indicate that antibodies against both EscC and LomW allow inhibition of EHEC early events during adhesion, prior to the intimate attachment to IECs and the subsequent pathogenic process.

Despite differences in the pathogenic mechanisms of the distinct E. coli pathogroups, there are shared homologous proteins linked to disease; therefore, the components that constitute these bacterial systems would be ideal antigens in the development of cross-protective vaccine candidates. Here, we describe the high sequence conservation of LomW between EAEC O104:H4 and EHEC, as well as of EscC between EPEC O127:H6 and EHEC (Table 1). Our cross-protective *in vitro* studies demonstrated inhibition of bacterial adherence to IECs as well as pedestal formation and cell cytotoxicity in EPEC and EAEC, respectively, and indicated that development of a vaccine that includes multiple strains of E. coli might be feasible. In the case of EAEC O104:H4, the characteristic aggregative adherence pattern and cell cytotoxicity (characterized by cell rounding and detachment from the substratum) are interrupted by serum from AuNP-LomW-immunized mice. For E. coli O127:H6 (EPEC), the level of the characteristic localized adherence pattern was reduced in the presence of AuNP-EscC; however, the formation of actin pedestals was not completely abolished. These results suggest variable effects associated with heterologous mechanisms in colonization, including the participation of additional pathotype-specific virulence factors. Further studies to increase the cross-protective effect with other pathotypes are needed to advance a multivalent vaccine.

Humoral responses play a major role in controlling extracellular pathogens, especially those enteric organisms that colonize the GI tract (40). Protective immune responses against EHEC are mediated by a Th2-biased humoral immune response that includes neutralizing IgA antibodies; however, IgG has also been shown to play a role (41, 42). The route of vaccination is important because the type and location (systemic versus mucosal) of the immune responses dictate the protective outcome of administration of a vaccine. Mucosal immunization against EHEC is associated with sIgA, although there have also been several candidates whose protection is correlated with IgG levels (34, 41, 42). Studies of systemic immunization routes, such as subcutaneous vaccination, have reported high levels of IgG as a protective mechanism in the absence of IgA (34). However, few vaccine candidates have been able to induce robust IgG and IgA responses. In our study, we demonstrated that subcutaneous immunization with the AuNP vaccine resulted in high-level serum IgG and fecal sIgA responses. Interestingly, the differences in the magnitudes of the IgG and IgA titers could have been the result of antibody fluctuations in fecal pellets or differences in the time of collection. In addition to the neutralization activity, we demonstrated the bactericidal properties of serum from AuNP-protein-immunized mice. We showed that antigen-specific serum from vaccinated mice damaged the bacterial membrane, thus indicating activation of the classical complement pathway. These results proved the functionality of serum antibodies in mediating bacterial killing. Previously, oral immunization with a live attenuated *Salmonella* strain expressing E. coli O157:H7 intimin was shown to elicit humoral and mucosal humoral responses (34). Our results corroborate some of those findings, including the involvement of both mucosal and systemic humoral responses in reducing intestinal colonization. However, we have now demonstrated that AuNPs elicit robust antigen-specific serum IgG and mucosal IgA responses without the need of a live attenuated vaccine platform.

In summary, we have demonstrated that our AuNP-vaccine platform can induce protective immune responses both *in vivo* and *in vitro* while coupling specific antigens targeting EHEC adhesion and colonization. We have also demonstrated the functionality of these immune responses *in vitro*. This subunit vaccine platform can induce strong humoral responses in response to each antigen as well as to the antigens in combination. We propose that conjugation of antigens on the surface of synthetic AuNPs is a viable approach for the design of effective bacterial vaccines.

## MATERIALS AND METHODS

### Bacterial strain and growth conditions.

The diarrheagenic Escherichia coli strains used in this study, EHEC (O157:H7), EPEC (O127:H6), and EAEC (O104:H4), were routinely grown aerobically at 37°C in Luria-Bertani (LB) broth. Before cell infection, the overnight cultures were diluted at 1:20 in Dulbecco’s minimum essential medium (DMEM) without fetal bovine serum (FBS) and antibiotics to express the type III secretion system components, as previously described (5). Bacterial cultures were incubated for 2 h at 37°C under conditions of static growth.

### Cloning.

EHEC (EDL933) DNA was isolated via the use of a Qiagen DNeasy blood and tissue kit, following the manufacturer’s directions. Sequences encoding LomW (GenBank protein accession no. 12514345) and EscC (GenBank protein accession no. 12518466) were amplified via the use of Phusion polymerase (New England BioLabs) and cloned into a pET30a(+) expression vector using NdeI and XhoI or HindIII-HF (New England BioLabs) restriction sites. The open reading frame for each protein was inserted in-frame with a 6His tag on the C terminus. Ligation, transformation, and expression experiments were performed according to directions of the manufacturer (pET System, Novagen) with some modifications. Upon confirmation of successful gene insertion via gel electrophoresis and directional sequencing (University of Texas Medical Branch [UTMB] 335 Genomics Core), plasmids were transformed into BL21(DE3) competent E. coli cells (New England 336 BioLabs) via heat shock treatment.

### Protein purification.

To induce protein expression, overnight cultures were diluted 1:20 in 1 liter of Luria Bertani (LB) broth, grown to an optical density at 600 nm (OD_600_) of between 0.6 and 0.8, and induced with 1 mM (final concentration) isopropyl β-d-1-thiogalactopyranoside (IPTG). Cultures were centrifuged (4,000 × *g* for 15 min) at 3 h postinduction, and each resulting bacterial pellet was frozen at –20°C. The bacterial pellets were then resuspended in 40 ml of phosphate-buffered saline (PBS) containing 10% glycerol and 25 mM sucrose with a 1 mg/ml final concentration of lysozyme and 0.2% sodium deoxycholate and a tablet of cOmplete EDTA-free protease inhibitor cocktail (Roche, Germany). This lysate was then sonicated and centrifuged, and the pellet was used for subsequent washes to maximize soluble protein extraction. After spin down, the supernatant was subjected to sterilization using a filter (0.2-μm pore size). Soluble protein extracts were then bound to Talon nickel columns (GE Healthcare, USA) and washed with PBS buffer–50 mM imidazole. Proteins were eluted from affinity columns by applying a PBS buffer with 10% glycerol and 25 mM sucrose and 250 mM imidazole. Fractions were collected and pooled before being dialyzed overnight at 4°C. Endotoxin levels were tested using a Pierce LAL chromogenic endotoxin quantification kit (Thermo Fisher Scientific, USA) following the instructions of the manufacturer. The limit of detection for endotoxin is approximately 0.1 EU/ml of solution. The purified proteins and protein standards were subjected to a colorimetric bicinchoninic acid assay (BCA) in accordance with the manufacturer’s protocol and were then stored at −80°C until use. For protein visualization, 2 μg of protein was run on SDS-PAGE gel by electrophoresis. Protein bands were visualized by staining with Coomassie blue stain (Bio-Rad), or gels were transferred to a nitrocellulose membrane for Western blot analysis. A mouse anti-histidine antibody (1:5,000) was used and the reaction mixture incubated overnight at 4°C, and horseradish peroxidase (HRP)-conjugated rabbit anti-mouse IgG was used as a secondary antibody. Protein bands were visualized by adding ECL substrate (Thermo Fisher Scientific, USA), and the results were imaged on film.

### Coupling of protein candidates onto AuNPs.

Gold nanoparticles (AuNPs), 15 nm in diameter, were synthesized by the Turkevich method as previously described (24). Briefly, 1 mM gold (III) chloride trihydrate underwent a reduction reaction with 90 mM sodium citrate dihydrate. Particle size and shape was established by transmission electron microscopy (TEM). To stabilize the conjugation of soluble antigens onto the AuNP surface, 0.1 mM 16-mercaptohexadecanoic acid (16-MHDA) and 0.1% Triton X-100 were added to AuNPs. After 2 h of incubation, this solution was filtered with centrifugation (EMB Millipore AmiconTM Ultra-15, 30 kDa molecular weight cutoff [MWCO]), and the procedure was repeated to ensure coverage. To covalently link soluble protein, 20 μg per ml of nanoparticles was added in the presence of DMTMM [4-(4,6-dimethoxy-1,3,5-triazin-2-yl)-4-methyl-morpholinium chloride]. The reactions were carried out in 100 mM borate buffer to allow conjugation. Attachment of 16-MHDA and protein was confirmed by measuring plasmon resonance via UV-Vis spectroscopy as well as by SDS-PAGE.

### Animal studies.

Female 6-to-8-week-old BALB/c mice were purchased from Jackson Laboratories (Bar Harbor, ME, USA) and maintained in an animal biosafety level 2 (ABSL2) facility. Animals were housed in microisolator cages under pathogen-free conditions with food and water available *ad libitum* and maintained on a 12-h light cycle. All animal protocols were reviewed and approved by the Institutional Animal Care and Use Committee of the University of Texas Medical Branch (protocol no. 1007037B). To allow adequate acclimation, mice were housed within the animal facility for 1 week prior to experimentation.

### Immunization and challenge study.

To evaluate the protective immunogenicity of LomW and EscC coupled to an AuNP (AuNP-LomW or AuNP-EscC), anesthetized BALB/c mice (*n* = 6 per group) were inoculated subcutaneously (s.c.) three times at 2-week intervals. Animals received AuNP-LomW or AuNP-EscC or a combination vaccine (AuNP-combination). Each vaccine formulation contained 10 μg of protein (LomW or EscC) along with 500 μg Alhydrogel (InvivoGen, USA) and 10 μg of detoxified cholera toxin (CT) (Sigma). For the combination group (AuNP-combination), mice received equal parts AuNP-LomW and AuNP-EscC (5 μg each for 10 μg total protein concentration). Control groups received adjuvant alone. To evaluate antibody titers, blood was drawn retro-orbitally 2 weeks following the last boost. To isolate sera, blood was incubated for 30 min at room temperature (RT) to allow clotting and centrifuged (10,000 × *g* for 10 min). Sera was removed and stored at –80°C until use. Fecal samples were taken prior to vaccination (baseline) and 2 weeks after the last immunization. For assays requiring serum, the sera from all immunized animals (*n* = 8) were pooled and stored.

### Infection and bacterial colonization.

At 2 weeks after administering the last immunization, all mice were challenged with a dose of ∼3 × 10^9^ CFU of E. coli O157:H7 strain 86-24 via gavage (200 μl). Food was restricted 12 h before infection but was administered *ad libitum* throughout the remainder of the study. At 2 h prior to challenge, mice were injected intraperitoneally with cimetidine (Sigma) (50 mg/kg of body weight) to reduce stomach acidity. Fecal samples were collected daily for 7 days to assess bacterial shedding. Fecal pellets were homogenized in PBS, serially diluted, plated on MacConkey agar plates, and incubated at 37°C. To enumerate bacterial colonization in gastrointestinal tract, mice were euthanized, and ceca and large intestines were removed. Organs were homogenized in 1 ml PBS, serially diluted, and plated on MacConkey agar to quantify bacterial colonization. The bacterial limit of detection (LOD) was determined to be 10 CFU/organ.

### Detection of protein-specific antibodies.

Baseline serum and fecal samples from mice administered adjuvant only, AuNP-LomW, AuNP-EscC, and the AuNP-combination formulation were collected 7 days before prime and 14 days after the second boost. Whole blood was collected via retro-orbital bleeding and stored in Microvette tubes without anticoagulant. The sera were separated by centrifugation and stored at –80°C. Fecal pellets were suspended in 1 ml of PBS and centrifuged to remove fecal debris, and the supernatants were stored at –80°C until use. The protein-specific total IgG and IgA titers were determined by indirect enzyme-linked immunosorbent assay (ELISA). Briefly, a microplate (Costar, Cambridge, MA) was coated with LomW or EscC (1 μg/well) in a mixture with 1× Dulbecco’s phosphate-buffered saline (DPBS) (Corning, USA) and maintained at 4°C overnight. Wells were washed twice with washing buffer (0.05% Tween 20–1× DPBS) and then blocked with blocking buffer (0.05% Tween 20, 2% bovine serum albumin [BSA], 1× DPBS) at RT for 2 h. The blocked wells were washed twice before addition of sample diluent (1% BSA–0.05% Tween 20–1× DPBS). The sera were added to each top dilution well in triplicate, and 2-fold dilutions were performed following incubation at RT for 2 h. Diluted goat anti-mouse IgG (Southern Biotech, USA) (1:5,000) was added into each well and then incubated for 3 h after washing. Plates were washed four times prior to addition of tetramethylbenzidine (TMB) substrate solution (Invitrogen, USA). Stop solution (2N H_2_SO_4_) was added, and the samples were immediately read at 450 and 570 nm using a microplate reader (BioTek, USA). The results were reported as the reciprocal of the highest titer, giving an optical density (OD) reading of at least the mean +2 standard deviations compared to the baseline sera. All assays were performed in triplicate, and results were reported as mean reciprocal endpoint titers.

### Adherence inhibition by serum assay.

Caco-2 cells (ATCC HTB-37) were maintained at 37°C with 5% CO_2_ in complete HTB-37 medium. Complete Dulbecco’s minimum essential medium (DMEM, Gibco, USA) supplemented with 2 mM glutamine, 1 mM sodium pyruvate, 1× nonessential amino acids, penicillin and streptomycin (100 U/ml and 100 μg/ml, respectively), and 10% fetal bovine serum. For adhesion assays, 12-well plates were seeded with 1 × 10^6^ cells/per well. At approximately 1 h prior to infection, the monolayer was washed twice with 1 ml PBS prior to addition of 1 ml medium containing no supplements. Overnight cultures of EHEC O157:H7 strain 86-24 were diluted in DMEM (1:20) without supplements and incubated at 37°C under static growth conditions for 2 h. Bacterial inocula were adjusted to an MOI of 10 (1 × 10^7^ CFU) and incubated in the presence of AuNP-LomW, AuNP-EscC, AuNP-combination immune serum, adjuvant-only serum, or naive sera (10%) for 1 h at 37°C with slight agitation. After incubation in the presence of sera, bacteria were collected in 1 ml of fresh media and used to infect cell culture plates containing 1 × 10^6^ cells. Monolayers were incubated for 2 h at 37°C with 5% CO_2_. After incubation, cells were washed three times with PBS prior to addition of 100 μl of 0.1% Triton X-100–PBS. After detachment, the cells were serially diluted in PBS and plated on LB agar plates for enumeration of adhered bacteria (output). The percentage of adhered bacteria was determined as the output/input × 100. Data represent results of two independent experiments performed using pooled sera from *n* = 8 mice.

### Serum bactericidal assay.

Serum bactericidal assays were done as previously described with modifications (43, 44). Sera from immunized animals (*n* = 8) were pooled and were either stored at –80°C or subjected to heat inactivation (56°C for 30 min). Serum from naive BALB/c mice was added to inactivated serum as an active source of complement. Bacterial cultures were grown as described above. Overnight EHEC 86-24 cultures were diluted 1:20 in DMEM without supplements and activated under static growth conditions for 2 h. Once the bacteria reached an OD_600_ of 0.5, 50-μl reaction mixtures containing 1 × 10^6^ CFU were prepared with 10% active, inactive, or inactive serum along an exogenous source of complement. Bacteria were incubated in the presence of serum for 1 h at 37°C with slight agitation. Viable CFU counts were determined by plating on LB agar plates at 1 h postincubation in mouse serum. The negative control contained bacteria with serum from mice immunized with adjuvant only. The serum bactericidal percentage was determined using the following equation: (bacterial CFU in adjuvant-only sera – bacterial CFU in treatment group sera)/bacterial CFU in adjuvant-only sera × 100. The results are obtained from two independent experiments using pooled sera from *n* = 8 mice.

### Fluorescence microscopy.

For microscopy fluorescence analysis, Caco-2 cells were cultured in round coverslips placed at the bottom of 12-well plates and incubated for 24 h at 37°C with 5% CO_2_. Bacterial inocula used at an MOI of 10 (1 × 10^6^ CFU) were incubated in the presence of AuNP-LomW, AuNP-EscC, or naive sera (10%) for 1 h at 37°C with slight agitation. After incubation in the presence or absence of sera, bacteria were collected in 1 ml of fresh media and used to infect cell culture plates containing 1 × 10^5^ cells. Additionally, evaluation of bacterial viability was performed by using LIVE/DEAD staining (LIVE/DEAD *Bac*Light bacterial viability kit; Invitrogen, USA) in accordance with the manufacturer’s instructions. For cell infection, monolayers were incubated for additional 2 h at 37°C with 5% CO_2_. After infection, cells were washed and fixed with 4% paraformaldehyde–PBS. Polymerized actin was detected by staining with tetramethyl rhodamine isothiocyanate-phalloidin (Molecular Probes-Invitrogen, USA). Cell nuclei and bacteria were mounted using Fluoroshield and DNAs detected with DAPI. EHEC (86-24) was detected by immunofluorescence with anti-E. coli O plus E. coli K antibody coupled to fluorescein isothiocyanate (FITC) (Abcam, USA). Images were taken using an Olympus BX51 upright fluorescence microscope and analyzed by the use of Image J software (University of Wisconsin-Medicine).

### Statistical analysis.

All statistical analysis was done using GraphPad Prism software (v 6.0). *P* values of <0.05 were considered statistically significant. Quantitative data are expressed as means ± standard errors. All data were analyzed for normality before the corresponding test was run. Results of colonization, antibody, serum adherence inhibition, and bactericidal assays were analyzed by one-way analysis of variance (ANOVA) (Tukey's *post hoc* test) or the Kruskal-Wallis test when data were not normally distributed.
