# Supplementary figures and images for: Development of a Gold Nanoparticle Vaccine against Enterohemorrhagic Escherichia coli O157:H7
Source: mBio. 2019 Aug 13;10(4):e01869-19. doi: 10.1128/mBio.01869-19 (PMC6692519; doi:10.1128/mBio.01869-19)

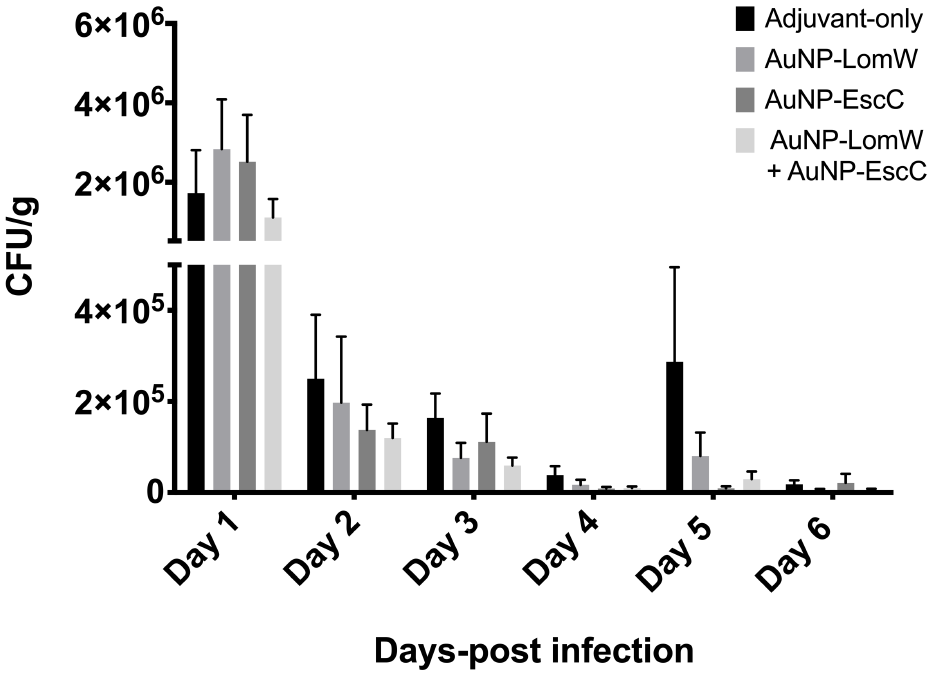

Supplement: FIG S1 [file mBio.01869-19-sf001.tif]
